# Dual-targeted CAR T-cell immunotherapies for hematological malignancies: latest updates from the 2023 ASH annual meeting

**DOI:** 10.1186/s40164-024-00485-8

**Published:** 2024-02-27

**Authors:** Jingyi Yang, Hao Guo, Lu Han, Yongping Song, Keshu Zhou

**Affiliations:** 1 Department of Hematology, the Affiliated Cancer Hospital of Zhengzhou University & Henan Cancer Hospital, Zhengzhou, 450008 China; 2 School of Life Sciences, Tsinghua University, Beijing, 100084 China; 3 Department of Immunology, the Affiliated Cancer Hospital of Zhengzhou University & Henan Cancer Hospital, Zhengzhou, 450008 China; 4 Department of Hematology, The First Affiliated Hospital of Zhengzhou University, Zhengzhou, 450052 China

**Keywords:** Chimeric antigen receptor, Dual-targeted, Hematological malignancy, Clinical trial

## Abstract

Over the past few years, dual-targeted chimeric antigen receptor (CAR) T-cell therapy has been employed in the management of hematological malignancies to mitigate treatment failure, particularly in cases of antigen escape. The most widely used approaches include CD19/CD20, CD20/CD22, and BCMA/CD19 CAR T-cells. Alternative immune cells, including natural killer T cells and invariant natural killer T cells, exhibit innate anti-tumor activity and reduced toxicity. This review summarizes several recent clinical trial reports and preclinical studies from the 2023 American Society of Hematology (ASH) annual meeting on dual-targeted CAR T-cell immunotherapy for hematological malignancies.

## To the editor

Although single-targeted chimeric antigen receptor (CAR) T cells have achieved remarkable success in treating hematological malignancies, relapse remains a major obstacle to achieving long-term survival [1]. CAR T cells targeting multiple antigens have been demonstrated to be an effective strategy to overcome antigen escape [2]. Here, we summarize the latest reports on dual-targeted CAR T-cell immunotherapy for hematological malignancies from the 2023 American Society of Hematology (ASH) annual meeting.

### Dual-targeted CAR T-cell therapies for B-NHL

Three trials reported the outcomes of CD19/CD20 CAR T-cell therapy for relapsed or refractory (r/r) B-cell non-Hodgkin lymphoma (B-NHL) [3–5]. Eleven r/r B-NHL patients (pts) received CART19/20 therapy derived from autologous naïve/memory T cells (Table 1) [3]. The overall objective rate (ORR) was 90.9% and the complete response (CR) rate was 72.7% (Table 2). With a median follow-up (mFU) of 32.3 months, the median progression-free survival (mPFS) and median overall survival (mOS) were not reached. Six pts experienced grade 1 cytokine release syndrome (CRS). No immune effector cell-associated neurotoxicity syndrome (ICANS) occurred.

LV20.19 CAR T cells, which also target CD20/CD19, are enriched for stem cell-like memory and central memory T cells (Table 1) [4]. A total of 17 pts with r/r mantle cell lymphoma (MCL) were enrolled, resulting in 100% ORR and 76% CR (Table 2). Two pts relapsed at 8 and 24 months, respectively. The 1-year PFS rate was 77%, and the 1-year OS rate was 84%. Two pts developed grade 3 ICANS. No severe CRS (grade ≥ 3) was observed.

C-CAR039, an autologous CD19/CD20 CAR T-cell therapy, was administered to r/r B-NHL pts resistant to CD20 antibody (Table 1) [5]. In total 91.5% of pts (43/47) achieved ORR, with 85.1% (40/47) achieving CR (Table 2). With a mFU of 23.9 months, the estimated 2-year PFS and OS were 66.0% and 77.9%, respectively. Only one patient experienced grade 3 CRS. No severe ICANS occurred.

ATA3431, an allogeneic CD19/CD20 CAR T-cell product, demonstrated enhanced persistence and superior anti-tumor activity in a mouse model of Burkitt’s lymphoma (Table 1) [6].

API-192, targeting CD19 and CD20, is a cord blood CD34 hematopoietic stem and progenitor cell-derived natural killer T (NKT) cell product armored with IL-15 (Table 1) [7]. API-192 exhibited robust expansion and serial tumor killing against Raji and Nalm6 cells.

UCART20 × 22 is the first human allogeneic CD20/CD22 CAR T-cell product (Table 1) [8]. All three r/r B-NHL pts treated with UCART20 × 22 demonstrated an objective response with two achieving CR. No ICANS, graft-versus-host disease (GvHD) or severe CRS occurred (Table 2).

Eight pts with r/r diffuse large B-cell lymphoma (DLBCL) were enrolled in the treatment of CD19/CD70 CAR T-cell therapy (Table 1) [9]. ORR was achieved in 87.5% of pts, with 75.0% evaluated as CR (Table 2). With a mFU of 19.9 months, three pts relapsed. The median disease-free survival (mDFS) was 10.5 months. Severe CRS or ICANS was not observed.

### Dual-targeted CAR T-cell therapies for ALL

Invariant natural killer T (iNKT) cells possess the ability to safeguard against GvHD without TCR deletion (Table 1). Allogeneic CD19/CD133 CAR iNKT-cell therapy exhibited remarkable efficacy in treating MLL-rearranged acute lymphoblastic leukemia (ALL), while also potentially preventing immune escape, leptomeningeal disease, and lineage switch [10].

### Dual-targeted CAR T-cell therapies for MM

A phase I clinical trial reported updated results of BCMA/CD19 CAR T-cell therapy (GC012F) for pts with newly diagnosed multiple myeloma (MM) (Table 1) [11]. The ORR was 100% and the stringent CR rate was 95.5% (Table 2). The mOS and mPFS were not reached with a mFU of 13.6 months. No ICANS or severe CRS was reported.

### Dual-targeted CAR T-cell therapies for AML

CLEC12a/TIM3 CAR T cells demonstrated potent anti-leukemic efficacy and exhibited prolonged persistence in the blood for 20 weeks following administration in an acute myeloid leukemia (AML) mouse model [12].

In summary, dual-targeted CAR T-cell immunotherapies have shown promising outcomes. Severe CRS or ICANS have not been reported in most clinical trials. Larger phase II trials with extended follow-up are necessary to determine whether these approaches can mitigate the risk of antigen loss/dim, relapse, and enhance the duration of response (DOR). Furthermore, dual-targeted immunotherapies derived from allogeneic NKT or iNKT cells have demonstrated the feasibility, potency, and safety necessary for further clinical validation.

**Table 1:** The properties of dual-targeted CAR T cells :

Product	Targets	Costimulatory domain	CAR generation	Transduction	Source of T cells	Refs.
CART19/20	CD19/CD20	4-1BB	Second	Lentiviral	Autologous T_N/MEM_ cells	[3]
LV20.19	CD19/CD20	4-1BB	Second	Lentiviral	Autologous T cells with T_SCM_ and T_CM_ phenotype	[4]
C-CAR039	CD19/CD20	4-1BB	Second	NA	Autologous T cells	[5]
ATA3431	CD19/CD20	NA	NA	NA	Allogeneic T cells from healthy donor with T_CM_ phenotype	[6]
API-192	CD19/CD20	NA	Fourth, armored with IL-15	NA	Human cord blood CD34 hematopoietic stem and progenitor cell-derived NKT cells	[7]
UCART20 × 22	CD20/CD22	NA	NA	Lentiviral	Allogeneic T cells from healthy donor	[8]
CD19/CD70 CAR T cells	CD19/CD70	CD28/CD27	Third	Lentiviral	Autologous T cells	[9]
CD19/CD133 CAR T cells	CD19/CD133	CD19: CD28 CD133: 4-1BB	Second	NA	Allogeneic iNKT cells from healthy donor	[10]
GC012F	BCMA/CD19	NA	NA	NA	Autologous T cells	[11]
TIM3/CLEC12a CAR T cells	TIM3/CLEC12a	CD28/4-1BB	Third	Retroviral	Autologous T cells	[12]

T_N/MEM_, naïve/memory T; T_SCM_, stem cell-like memory T; T_CM_, central memory T; NKT, natural killer T; iNKT, invariant natural killer T; NA, not available

**Table 2:** Outcomes of clinical trials of dual-targeted CAR T-cell immunotherapies in hematological malignancies from ASH 2023

Product	CART19/20	LV20.19	C-CAR039	UCART20 × 22	CD19/CD70 CAR T-cell therapy	GC012F
Disease	r/r B-NHL	r/r MCL	r/r B-NHL	r/r B-NHL	r/r DLBCL	NDMM
Clinical trial phase	I	I/II	Ib/II	I/IIa	I/II	I
Targets	CD19/CD20	CD19/CD20	CD19/CD20	CD20/CD22	CD19/CD70	BCMA/CD19
Dosage	50 × 10^6^ ± 30% cells 200 × 10^6^ ± 30% cells	2.5 × 10^6^cells/kg	1.0–5.0 × 10^6^cells/kg	50 × 10^6^ cells	NA	1 × 10^5^ cells/kg 2 × 10^5^ cells/kg 3 × 10^5^ cells/kg
Patients (n)	11	17	48	3	8	22
ORR	90.9%	100% at day 28 100% at day 90	91.5%	100.0%	87.5%	100.0%
CR	72.7%	76% at day 28 92% at day 90	85.10%	66.7%	75.0%	95.5%
mFU (months)	32.3	14	23.9	NA	19.9	13.6
Relapse	NA	2 pts	NA	NA	3 pts	NA
Survival	mPFS: NR mOS: NR	PFS: 77% at 1-year OS: 84% at 1-year DOR: 92% at 1-year	PFS: 66.0% at 2-year OS: 77.9% at 2-year	NA	mDFS: 10.5 months mOS: NR	mPFS: NR mDOR: NR
CRS	54.50%	94.1%	93.80%	100%	37.50%	27.30%
Grade 1–2	54.50%	94.1%	91.70%	100%	37.50%	27.30%
Grade 3–4	0%	0%	2.10%	0%	0%	0%
ICANS	0%	17.60%	6.30%	0%	0%	0%
Grade 1–2	0%	5.90%	6.30%	0%	0%	0%
Grade 3–4	0%	11.80%	0%	0%	0%	0%
Clinical trial number	NCT04007029	NCT04186520	NCT04693676	NCT05607420	NCT05436496	NCT04935580
References	[3]	[4]	[5]	[8]	[9]	[11]

ORR, overall objective rate; CR, complete response; mFU, median follow-up; mDFS, median disease-free survival; mPFS, median progression-free survival; mOS, median overall survival; mDOR, median duration of response;CRS, cytokine release syndrome; ICANS, immune effector cell-associated neurotoxicity syndrome; r/r, relapsed or refractory; B-NHL, B-cell non-Hodgkin lymphoma; MCL, mantle cell lymphoma; DLBCL, diffuse large B-cell lymphoma; ND, newly diagnosed; MM, multiple myeloma; pts, patients; NR, not reached; NA, not available

## Data Availability

No datasets were generated or analysed during the current study.

## Abbreviations

ASH: American Society of Hematology
ALL: Acute lymphoblastic leukemia
AML: Acute myeloid leukemia
B-NHL: B-cell non-Hodgkin lymphoma
CAR: Chimeric antigen receptor
CR: Complete response
CRS: Cytokine release syndrome
DLBCL: Diffuse large B-cell lymphoma
DOR: Duration of response
GvHD: Graft-versus-host disease
ICANS: Immune effector cell-associated neurotoxicity syndrome
iNKT: Invariant natural killer T
MCL: Mantle cell lymphoma
MM: Multiple myeloma
mDFS: Median disease-free survival
mFU: Median follow-up
mOS: Median overall survival
mPFS: Median progression-free survival
NA: Not available
ND: Newly diagnosed
NKT: Natural killer T
NR: Not reached
ORR: Overall objective rate
pts: Patients
r/r: Relapsed or refractory
T_CM_: Central memory T
T_N/MEM_: Naïve/memory T
T_SCM_: Stem cell-like memory T
